# Smoking decreases the response of human lung macrophages to double-stranded RNA by reducing TLR3 expression

**DOI:** 10.1186/1465-9921-14-33

**Published:** 2013-03-09

**Authors:** Jill C Todt, Christine M Freeman, Jeanette P Brown, Joanne Sonstein, Theresa M Ames, Alexandra L McCubbrey, Fernando J Martinez, Stephen W Chensue, James M Beck, Jeffrey L Curtis

**Affiliations:** 1 Division of Pulmonary & Critical Care Medicine, Department of Internal Medicine, University of Michigan Health Care System, Ann Arbor, MI, 48109-2399, USA; 2 Research Service, Department of Veterans Affairs Health Care System, Ann Arbor, MI, 48105-2303, USA; 3 Graduate Program in Immunology, University of Michigan Health Care System, Ann Arbor, MI, 48109-2399, USA; 4 Department of Pathology, University of Michigan Health Care System, Ann Arbor, MI, 48109-5602, USA; 5 Pathology & Laboratory Medicine Service, Department of Veterans Affairs Health Care System, Ann Arbor, MI, 48105-2303, USA; 6 Pulmonary & Critical Care Medicine Section, Medical Service, Department of Veterans Affairs Health Care System, 2215 Fuller Road, Ann Arbor, MI, 48105-2303, USA

**Keywords:** Lung, Cigarette smoking, Effects, Toll-like receptors, Macrophages, Alveolar

## Abstract

**Background:**

Cigarette smoking is associated with increased frequency and duration of viral respiratory infections, but the underlying mechanisms are incompletely defined. We investigated whether smoking reduces expression by human lung macrophages (Mø) of receptors for viral nucleic acids and, if so, the effect on CXCL10 production.

**Methods:**

We collected alveolar macrophages (AMø) by bronchoalveolar lavage of radiographically-normal lungs of subjects undergoing bronchoscopies for solitary nodules (*n* = 16) and of volunteers who were current or former smokers (*n* = 7) or never-smokers (*n* = 13). We measured expression of mRNA transcripts for viral nucleic acid receptors by real-time PCR in those AMø and in the human Mø cell line THP-1 following phorbol myristate acetate/vitamin D3 differentiation and exposure to cigarette smoke extract, and determined TLR3 protein expression using flow cytometry and immunohistochemistry. We also used flow cytometry to examine TLR3 expression in total lung Mø from subjects undergoing clinically-indicated lung resections (*n* = 25). Of these, seven had normal FEV1 and FEV1/FVC ratio (three former smokers, four current smokers); the remaining 18 subjects (14 former smokers; four current smokers) had COPD of GOLD stages I-IV. We measured AMø production of CXCL10 in response to stimulation with the dsRNA analogue poly(I:C) using Luminex assay.

**Results:**

Relative to AMø of never-smokers, AMø of smokers demonstrated reduced protein expression of TLR3 and decreased mRNA for TLR3 but not TLR7, TLR8, TLR9, RIG-I, MDA-5 or PKR. Identical changes in TLR3 gene expression were induced in differentiated THP-1 cells exposed to cigarette smoke-extract in vitro for 4 hours. Among total lung Mø, the percentage of TLR3-positive cells correlated inversely with active smoking but not with COPD diagnosis, FEV1% predicted, sex, age or pack-years. Compared to AMø of never-smokers, poly(I:C)-stimulated production of CXCL10 was significantly reduced in AMø of smokers.

**Conclusions:**

Active smoking, independent of COPD stage or smoking duration, reduces both the percent of human lung Mø expressing TLR3, and dsRNA-induced CXCL10 production, without altering other endosomal or cytoplasmic receptors for microbial nucleic acids. This effect provides one possible mechanism for increased frequency and duration of viral lower respiratory tract infections in smokers.

**Trial registration:**

ClinicalTrials.gov NCT00281190, NCT00281203 and NCT00281229.

## Background

Viral respiratory infections are major risk factors for exacerbations of chronic obstructive pulmonary disease (COPD) and asthma [1,2], contributing to enormous societal costs due to healthcare utilization and reduced productivity. The frequency and duration of viral respiratory infections is increased by cigarette smoking [3-6] through multiple mechanisms, including reduced mucociliary clearance, impaired production of epithelial defensins, and decreased neutrophil chemotaxis {reviewed in [7,8]}. Smoking also negatively impacts host defense by alveolar macrophages (AMø), a key phagocyte and source of inflammatory mediators. Acting via both nicotinic cholinergic receptors [9] and uncharacterized mechanisms [10], smoking decreases pro-inflammatory cytokine release by human AMø [11-13]. Cigarette smoke extract (CSE) also blunts the ability of AMø to be activated by IFN-γ produced by T cells and natural killer (NK) cells [14]. Whether smoking has additional effects predisposing to viral respiratory infections is unknown.

We hypothesized that cigarette smoke could also reduce the ability of AMø to detect and activate innate responses to respiratory viruses. Cells recognize viruses by sensing double-stranded (ds) RNA using TLR3, which is expressed on endosomes and in some cell types, on the cell surface [15,16]. The other endosomal microbe-associated molecular pattern (MAMP) receptors, TLR7, TLR8 and TLR9, are also important for recognizing single stranded RNA and bacterial DNA with unmethylated CpG dinucleotides, respectively. Alternatively, dsRNA can also be recognized via cytosolic sensors, such as double-stranded RNA-dependent protein kinase R (PKR), retinoic acid-inducible gene I (RIG-I), or melanoma differentiation-associated gene 5 (MDA-5) [17].

Experimentally, the response to dsRNA is measured using the synthetic analogue polyinosinic acid:cytidylic acid (poly(I:C)), which stimulates secretion of the chemokine CXCL10 (formerly IP-10) [18-20], in some cell types in an entirely TLR3-dependent fashion [21]. CXCL10 is important to recruit and activate neutrophils, lymphocytes and NK cells [22-24], which are crucial to limit viral replication. At concentrations 100-fold higher than needed for chemotactic activity, CXCL10 also has defensin-like antimicrobial activities [25] and can enhance Mø killing of intracellular *Leishmania*[26].

The purpose of this study was to determine the effect of smoking on endosomal and cytoplasmic receptors for nucleic acids. Our results demonstrate the susceptibility in human AMø of TLR3, but not of other receptors tested, to smoke-induced down-regulation.

## Methods

### Research subjects

Studies and consent procedures were performed in accordance with the Declaration of Helsinki at the VA Ann Arbor Healthcare System (all bronchoscopies and some clinically-indicated lung resections) and the University of Michigan Health System (some clinically-indicated lung resections, including all lung transplants and lung volume reduction surgeries) and were approved by their Institutional Review Boards (FWA 00000348 & FWA 00004969, respectively). All subjects understood the purpose of the study and gave written consent before any research procedures. All subjects underwent a complete history and physical examination by a Pulmonologist, spirometry, chest imaging, prospective collection of medication history, and complete blood count with differential, coagulation studies and chemistry panel.

### Bronchoalveolar lavage (BAL) cohort

We recruited 13 healthy never-smoker volunteers and 23 subjects with a smoking history who either were scheduled to undergo bronchoscopy in the evaluation of solitary pulmonary nodules (*n =* 16) or who volunteered (*n* = 7). Among the 23 subjects who had ever smoked, 18 were active smokers (<six months since quitting) and 5 were former smokers (>six months since quitting). Of those with any smoking history, subjects (*n* =10) with ≥10 pack years, a ratio of forced expiratory volume in 1 second to forced vital capacity (FEV_1_/FVC) >0.7, normal spirometry, and no clinical diagnosis of COPD represent control smokers. Subjects (*n* =13) with a smoking history, FEV_1_/FVC <0.7 and abnormal spirometry were considered to have COPD. All subjects were without evidence of lung infection, interstitial lung disease or collagen vascular disease.

Importantly, not all types of experiments were performed on cells from every subject in this cohort, and conversely, some subjects were used for more than one type of experiment. The characteristics of the BAL subjects used in each type of experiments are summarized in Tables in the Results sections; demographic and clinical data for this entire cohort are shown in Additional file 1: Table S1.

### Lung tissue cohort

Lung tissue was collected from consented subjects undergoing clinically-indicated resections for pulmonary nodules, lung volume reduction surgery, or lung transplantation (*n* = 25). Using the same definitions as in the BAL cohort, this cohort comprised seven control smokers and 18 subjects with COPD. This cohort was used exclusively for flow cytometric analysis of total lung Mø. Characteristics of this cohort are summarized in the Results section, and demographic and clinical data for individual subjects are shown in Additional file 2: Table S2.

### BAL procedure and cell preparation

BAL was performed in the right middle lobe and lingula of the volunteers and in whichever of these sites was contralateral to the nodule in the clinically-indicated bronchoscopies, in which the research BAL was performed as early as feasible, and always before any biopsies or brushings. We instilled 100 ml normal saline per site, using a 30 ml syringe and gentle manual suction. BAL fluid was filtered through sterile gauze to remove mucous, and cells were washed thrice with PBS, with centrifugation between washes.

BAL cells (>95% AMø by Wright-Giemsa-stained cytospins) were either immediately processed for flow cytometry and immunohistochemistry or were cultured briefly to purify AMø by adherence. For this purpose, cells were resuspended in complete medium (RPMI 1640 containing 25 mM HEPES, 2 mM l-glutamine, 1 mM pyruvate, 100 U/ml penicillin/streptomycin, 10% heat-inactivated AB human serum (all from Invitrogen, Carlsbad, CA), and 55 μM 2-ME (Sigma Chemical, St. Louis, MO), and were plated at 2 × 10^5^ cells/well in sterile 24-well plates. Cells were incubated for 1.5 h in 5% CO_2_ at 37°C, washed to remove nonadherent cells, then incubated in AIM-V serum-free medium (Invitrogen) under experimental conditions. For TLR3 stimulation, adherence-purified AMø were cultured in AIM-V alone or with poly(I:C) at 50 μg/ml for 24 h.

### Lung sample preparation

Lung tissue was dissected free of any areas containing nodules, cancers or evidence of infection by a Pathologist before being released to the study. Lung sections weighing approximately 3 g were dispersed using a Waring blender in a biosafety cabinet without enzyme treatments, which we have previously shown produces single cell suspensions of high viability and functional capacity [27,28]. Cells were filtered through a 40 μm strainer to remove debris and were resuspended in staining buffer (2% FBS in PBS) for flow cytometery.

### Differentiation and treatment of THP-1 cells

THP-1 cells obtained from ATCC (Manassas, VA) were grown according to the supplier’s guidelines. Cells were plated at 4 × 10^5^ cells/well in 24-well plates, and after adherence, were differentiated by exposure to 150 ng/ml phorbol myristate acetate (PMA) and 0.01 μM vitamin D3 for 48 hrs, and then were washed with fresh complete medium before exposure to various concentrations of cigarette smoke-extract (CSE) for 4 hr. Fresh CSE was prepared by bubbling the mainstream smoke of two University of Kentucky Tobacco Health Research Institute standardized cigarettes (lot 2R4F), using a Jaeger-Baumgartner Cigarette Smoking Machine (C.H. Technologies, New Jersey) driven by dry compressed air, into 20 ml RPMI 1640; the result was defined as 10% CSE. CSE was filter-sterilized using a 0.22 μm membrane and was used the same day.

### Quantitative real-time PCR

RNA was isolated using RiboPure kits (Applied Biosystems, Foster City, CA), TURBO DNA-free (Applied Biosystems) to remove genomic DNA, and Retroscript kits (Applied Biosystems) for reverse-transcription. We performed quantitative real-time PCR using the Stratagene Mx3000P (LaJolla, CA), with human glyceraldehyde-3-phosphate dehydrogenase (GAPDH) as the endogenous reference, and commercial primer-probe sets (Taqman chemistry, Applied Biosystems). Transcript levels were reported relative to a calibrator of unstimulated THP cells, or AMø from never smokers, as appropriate, as calculated on the thermocycler.

### Flow cytometry

BAL cells were resuspended in 100 μL staining buffer {1% FA Buffer (BD Bioscience, San Jose, CA), 1% FCS, 0.01% sodium azide} per tube. We used antibodies against the following antigens (clones in parentheses): CD45 (2D1) (eBioscience, San Diego, CA), TLR3 (40C1285.6), TLR7 (polyclonal), TLR9 (26C593.2) (Imgenex Corp., San Diego, CA), and TLR8 (303F1.14) (Dendritics, Lyon, France). To detect intracellular receptor expression, cells were treated with Fixation and Permeabilization Buffers (eBioscience). In all experiments, we used isotype-matched controls, analyzed cells on an LSR II flow cytometer (BD Bioscience), and collected a minimum of 10,000 CD45+ events per sample, using FACS Diva software with automatic compensation, and FlowJo analysis software (Tree Star, Inc., Ashland, OR). Details of instrument setup have been described recently [29].

### Immunohistochemistry

Cytospins were immersed in cold acetone followed by methanol plus hydrogen peroxide. The slides were stained with biotinylated anti-human TLR3 (Imgenex) or isotype control antibodies, followed by Universal ABC peroxidase complex and 3-amino-9-ethylcarbazole peroxidase substrate. Slides were counter-stained with hematoxylin and photographed using an Olympus BX51 digital camera.

### CXCL10 analysis

CXCL10 levels were determined using Biosource Multiplex Assays (Invitrogen) and a Luminex 200™ (Luminex Corporation, Austin, TX).

### Statistical analyses

Statistical analysis was performed using GraphPad Prism (GraphPad, La Jolla, CA) except as specified. We used the nonparametric unpaired Mann–Whitney test to determine differences between two groups (smokers versus never-smokers; healthy subjects versus COPD subjects) in mRNA and flow cytometry data; the nonparametric Kruskal-Wallis one-way ANOVA to compare multiple conditions on THP cells in vitro; and ANOVA with Bonferroni’s Multiple Comparison post-hoc testing to determine differences between smokers and never-smokers in stimulated cytokine secretion. We used Spearman non-parametric analysis to correlate TLR3 mRNA and protein expression with clinical variables and performed linear regression using SPSS (IBM Corp.; Armonk, NY). A two-tailed *p* value of < 0.05 was considered significant.

## Results

### TLR3 expression is selectively decreased in AMø of smokers

**Figure 1:**
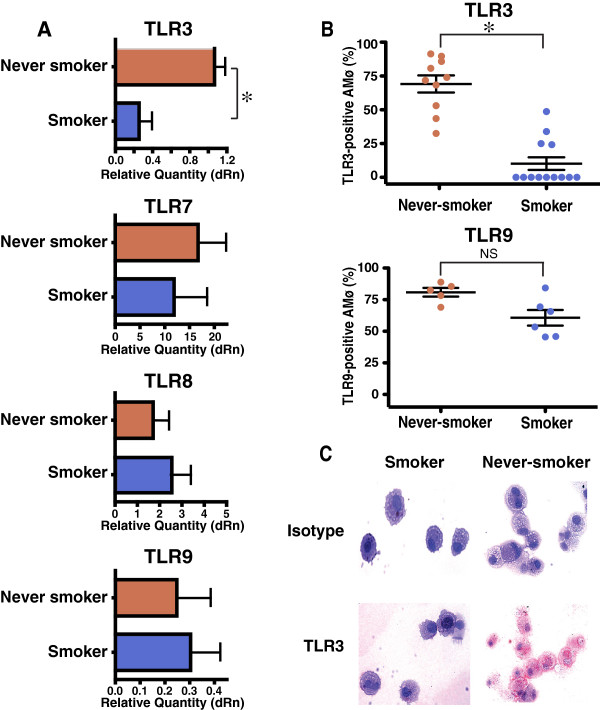
**AMø of current smokers show reduced expression of TLR3 mRNA transcripts and intracellular protein relative to AMø of never-smokers. A**. mRNA transcripts. RNA was isolated from AMø, depleted of contaminating genomic DNA, reverse-transcribed and analyzed by quantitative real-time RT-PCR using Taqman chemistry and specific primer-probe sets, normalized to GAPDH transcripts. Data are expressed on the horizontal axis as mean ± SEM for relative quantity (dRn), calculated in comparison to a single never-smoker who was arbitrarily designated the reference sample. Never-smokers (*n* = 6), red bars; smokers (*n* = 11), blue bars. *, *p* < 0.05 by Mann–Whitney test. **B**. Intracellular TLR3 (top panel) and TLR9 (bottom panel) expression by flow cytometry. AMø were permeabilized, stained for TLR3 and TLR9 expression and analyzed by flow cytometry. Data are expressed as TLR-positive AMø as mean ± SEM, never-smokers (*n* = 10 for TLR3, *n* = 5 for TLR9), red circles; smokers (*n* = 13 for TLR3, *n* = 6 for TLR9), blue circles. *, *p* < 0.05 by Mann–Whitney test. **C**. Immunohistochemical staining. Cytospins from a representative smoker (right) and a never-smoker (left), stained for intracellular TLR3 expression using AEC (red product) and hematoxylin (original 20 X). Top row, isotype control staining, bottom row, specific TLR3 staining. Representative of three experiments with similar results.

To test whether cigarette smoking impacts expression by human AMø of TLRs implicated in defense against respiratory viruses, we first analyzed adherence-purified AMø obtained by BAL of 11 current or former smoking subjects and six never-smokers (Table 1) using quantitative real-time PCR. Results showed significantly decreased TLR3 mRNA transcripts in smokers compared with never-smokers (p = 0.0015; Mann–Whitney test) (Figure 1A), but no significant differences in transcripts for TLR7, TLR8 or TLR9, which are also found in the endosome (Figure 1A). We also found no significant differences between smokers and never-smokers in mRNA transcripts for the cytoplasmic dsRNA receptors RIG-I, MDA-5 and PKR (Additional file 3: Figure S1).

**Table 1:** Characteristics of BAL subjects used for RNA studies

**Group**	**Smokers**	**Never-smokers**	***p *value**
Subjects, *n*	11	6	
Sex ratio, M/F	11/0	2/4	0.003
Age, years (SD)	65.5 (7.2)	37.7 (12)	0.002
Smoking, pack-years (SD)	65.5 (23.1)	0 (0)	0.0008
Smoking status (Former/Current)	2/9	0/0	0.003
FEV_1_, % pred. (SD)	57 (22.1)	101 (8.7)	0.002
FEV1/FVC% (SD)	58.5 (15.6)	86.8 (4.9)	0.0007
ICS use (yes/no)	7/4	0/6	0.02

*ICS* Inhaled Corticosteroid Use, *M* Male, *F* Female. Data are represented as mean (SD), ratio of current smokers to former smokers, or fraction of ICS users (yes/no). All FEV1 values are pre-bronchodilator. *p* values calculated using Mann Whitney test.

These findings were supported by two measures of AMø protein expression. Flow cytometry (Additional file 4: Figure S2) permitted identification of specific staining for TLR3 and TLR9 amongst AMø of never-smokers, although expression of TLR7 was very low (Additional file 4: Figure S2, middle panel) in both groups, and was not analyzed further. Comparing BAL samples from smokers (*n* = 13), and never-smokers (*n* = 10) (Table 2), we found a significantly decreased percentage (*p* < 0.0001, Mann–Whitney test) of AMø positive for intracellular TLR3 in AMø of smokers (Figure 1B). Note that these experiments contain some of the same subjects studied in Table 1, as well as other BAL subjects, as described in *Methods*; for additional details about individual subjects, see Additional file 1: Table S1). TLR3 was not detected on the surface of unpermeabilized AMø of either group (not shown). There were no differences between smokers (*n* = 6) and never-smokers (*n* = 5) in the percentage of AMø expressing intracellular TLR9 (Figure 1B) (or TLR7 and TLR8, data not shown). Similarly, immunohistochemical staining of BAL cytospins (Additional file 1: Table S1) demonstrated greatly reduced TLR3 expression in the AMø from smokers, relative to AMø from the never-smokers (Figure 1C). These independent data indicate that reduction in TLR3 protein expression measured by flow cytometry did not result simply from greater autofluorescence in AMø of smokers.

**Table 2:** Characteristics of BAL subjects used for flow cytometry studies

**Group**	**Smokers**	**Never-smokers**	***p *value**
Subjects, *n*	13	10	
Sex ratio, M/F	11/2	1/9	0.0006
Age, years (SD)	60.3 (8.9)	42.2 (13.7)	0.002
Smoking, pack-years (SD)	47.4 (33.7)	0 (0)	0.0001
Smoking status (Former/Current)	3/10	0/0	0.0001
FEV_1_, % pred. (SD)	83.5 (23.6)	100.7 (10.5)	0.077
FEV1/FVC% (SD)	72.5 (14.3)	84.2 (5.7)	0.0249
ICS use (yes/no)	4/9	0/10	N.S.

*ICS* Inhaled Corticosteroid Use; *M* Male, *F* Female. Data are represented as mean (SD), ratio of current smokers to former smokers, or fraction of ICS users (yes/no). All FEV1 values are pre-bronchodilator. *p* values calculated using Mann Whitney test.

**Figure 2:**
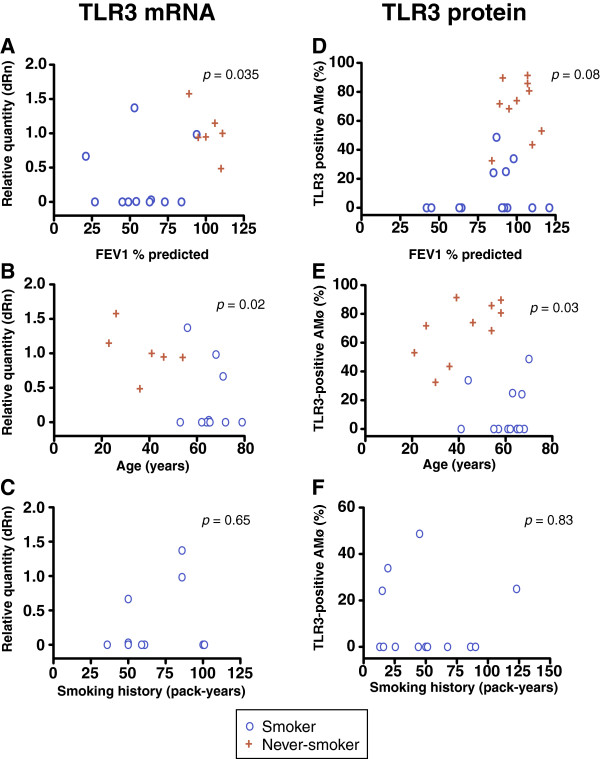
**AMø TLR3 expression does not correlate with lung function or total duration of smoking exposure.** AMø were harvested, processed and analyzed as described in the legend to Figure 1. **A**-**C**. TLR3 mRNA transcripts by quantitative real-time PCR (smokers, *n* = 11; never-smokers, *n* = 6, except in panel **C**, which includes only current or former smokers). **D**-**F**. Flow cytometric analysis of TLR3 protein expression (smokers, *n* = 13; never-smokers, n = 10, except in panel **F** which includes only current or former smokers). **A**, **D**: FEV1 % predicted; **B**, **E**: participant age (years); **C**, **F**: smoking history (pack-years); note the difference in the vertical scale of panel **F**. Blue circles, smokers; red crosses, never-smokers. *p* values by Spearman correlation.

In univariate analyses including both smokers and never-smokers, TLR3 RNA transcripts correlated inversely with both FEV_1_ % predicted and subject age (Figure 2A-B). Considering only those with a history of smoking, there was no correlation with pack-years (Figure 2C). However, in a linear regression, none of the variables (smoker versus never-smoker, age, sex, FEV_1_ % predicted) reached statistical significance for mRNA transcripts. AMø positivity for TLR3 protein did not correlate in univariate analyses with FEV_1_ % predicted (Figure 2D) or pack-years of smoking exposure (Figure 2F), but did correlate with subject age (Figure 2E). In a linear regression, smoking status (smoker vs. never-smoker) was strongly associated with TLR3+ AMø (*p* < 0.0001) and subject age was also significant (*p* = 0.035), but sex and FEV_1_ % predicted remained insignificant. When we analyzed smokers with COPD versus smokers without COPD, no significant differences were seen in TLR3 mRNA transcripts (COPD, 0.23 ± 0.16 vs. no COPD, 0.49 ± 0.49, mean ± SEM dRn; *p* = 0.52, unpaired *t* test) or flow cytometric results (COPD, 12.2 ± 12.2 vs. non-COPD, 10.4 ± 5.2; mean ± SEM % TLR3-positive AMø, *p* = 0.87, unpaired *t* test). Despite the use of inhaled corticosteroids (ICS) by ~50% of the smoking subjects, the decrease in the fraction of TLR3+ AMø of smokers was not a result of steroid usage (*p* = 0.79, determined by *t*-test comparing TLR3 mRNA transcript expression between ICS users and non-users; *p* = 0.92 when comparing TLR3 protein measurements by flow cytometry).

Thus, these secondary analyses supported a significant effect of smoking on AMø expression of TLR3. However, due to the distribution of our subjects, we were unable to exclude the possibility that our findings resulted from the preponderance of women in the never-smoker group and of men among the smokers in this cohort, or from the significant difference in ages between the volunteer and clinical subjects.

### Reduced TLR3 expression in lung Mø correlates with smoking status but not COPD stage

In apparent contradiction to our findings, a recent paper primarily using immunohistochemistry in lung tissue sections reported that the percentage of TLR3-positive lung Mø were significantly increased in smokers compared with never-smokers [30]. To address this discrepency and the questions about the possible effects of age and sex in our BAL subjects, we studied a different cohort of subjects (*n* = 25) undergoing clinically-indicated lung resection procedures (Table 3). This cohort also had the advantage of having more nearly balanced ratios of male and female subjects, as well as a wide range of spirometry values. We used mechanical disaggregation of tumor-free lung parenchyma to produce a single cell suspension of high viability containing both AMø and interstitial lung Mø, which we identified as autofluorescent, CD45+, high side scatter cells.

**Table 3:** Characteristics of surgical lung tissue subjects

**Group**	**Smokers with COPD**	**Smokers without COPD**	***p *value**
Subjects, *n*	18	7	
Sex ratio, M/F	10/8	5/2	0.49
Age, years (SD)	61.8 (9.4)	54.4 (9.1)	0.74
Smoking, pack-years (SD)	60.3 (41.4)	44.5 (23.9)	0.47
Smoking status (Former/Current)	4/14	3/4	0.11
FEV_1_, % pred. (SD)	41.8 (27.7)	95.4 (11.2)	0.0008
FEV1/FVC% (SD)	42.5 (18.6)	76.0 (5.2)	0.0002
ICS use (yes/no)	12/6	1/6	0.02

*ICS* Inhaled Corticosteroid Use, *M* Male, *F* Female. Data are represented as mean (SD), ratio of current smokers to former smokers, or fraction of ICS users (yes/no). All FEV1 values are pre-bronchodilator. *p* values calculated using Mann Whitney test.

**Figure 3:**
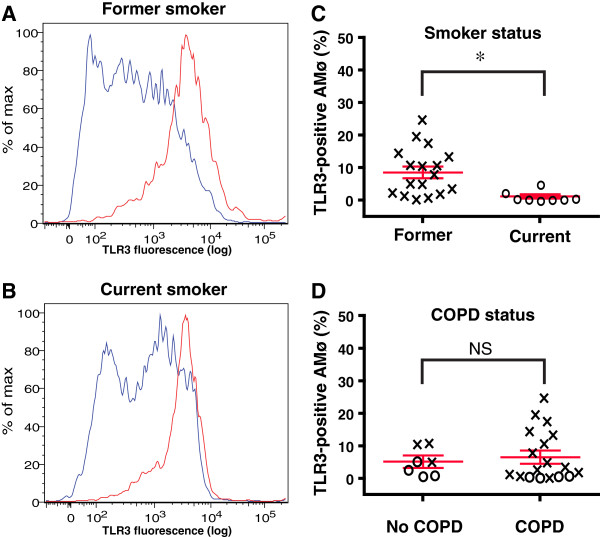
**In current smokers, the frequency of lung Mø expressing TLR3 protein is reduced relative to lung Mø of former-smokers.** To isolate total lung Mø, areas of human lung parenchyma, resected for clinical indications and remote from any evidence of nodules or infection, were processed by mechanical disaggregation without the use of enzymes. Lung Mø were permeabilized, stained for TLR3 expression and analyzed by flow cytometry. **A**, **B**. Representative histograms of intracellular TLR3 expression; **A**, former smoker; **B**, current smoker. **C**. Percentage of lung Mo expressing TLR3 relative to smoking status; x’s represent former smokers (*n* = 17); circles represent current smokers (*n* = 8). **D**. Percentage of TLR3-positive by lung Mø relative to COPD status; as in panel **C**, x’s represent former smokers (*n* = 17); circles represent current smokers (*n* = 8); *, *p* < 0.05; NS, non-significant. The Mann–Whitney test was used to calculate *p* values.

Flow cytometric analysis permitted objective quantification of the percentage of individual lung Møs positive for intracellular TLR3, relative to simultaneously analyzed isotype-control monoclonal antibodies (Figure 3A, B). We found the percentage of TLR3-positive lung Møs from current smokers was significantly reduced (p = 0.006) compared to the percentage of TLR3-positive lung Møs from former smokers (Figure 3C). By contrast, considering these same individuals, there were no significant differences in percentages of lung Mø expressing TLR3 between those with normal pulmonary function and those with COPD (Figure 3D). Nor was there a correlation between the percentage of TLR3-positive lung Mø by flow cytometry and either FEV1 % predicted or duration of smoking in pack-years (Additional file 5: Figures S3A & B). In a linear regression, smoking status (current vs. former) was significantly associated with TLR3 positivity (*p* = 0.016) and was not affected by age, FEV_1 %_ predicted, sex, or pack-years. These independent data agree with and extend results of our BAL experiments and collectively show that active smoking reduces TLR3 expression by resident human lung Mø.

### Exposure to CSE specifically reduces expression of TLR3 mRNA transcripts by differentiated human THP-1 cells

**Figure 4:**
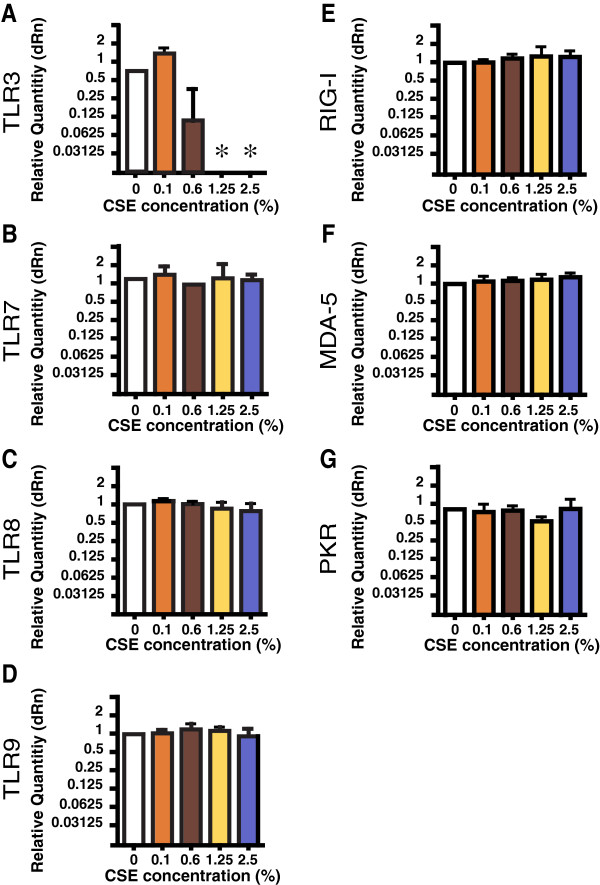
**Exposure to CSE specifically and rapidly reduces TLR3 gene expression in a differentiated human Mø cell line.** THP-1 cells differentiated using PMA & vitamin D3 for 48 hours were cultured in complete medium alone or with various concentration of CSE for an additional 4 hr, then analyzed by quantitative real-time RT-PCR. Data are mean ± SEM of four independent experiments, expressed as dRn (relative to differentiated THP-1 cells cultures in complete medium alone). **A**, TLR3; **B**, TLR7; **C**, TLR8; **D**, TLR9; **E**, RIG-1; **F**, MDA-5; **G**, PKR. Kruskal-Wallis one-way ANOVA was used to compare groups. *, *p* < 0.05, compared to cells that did not receive CSE exposure.

To explore the effect of acute smoke-exposure in vitro, we used the well-characterized system of differentiating the human Mø cell line THP-1 by treatment with PMA plus vitamin D3. In four independent experiments, we found that CSE at concentrations of 1.25% and 2.5% significantly decreased expression of TLR3 transcripts (Figure 4A), but had no effect on transcripts of TLR7, TLR8 or TLR9 (Figures 4B-D). CSE at these concentrations had no effect on viability as assessed by trypan blue exclusion (not shown). CSE also had no significant effect on mRNA transcripts for RIG-I, MDA-5 or PKR at concentrations up to 2.5% CSE (Figure 4E-G). Thus, the effect of smoke exposure on TLR3 mRNA levels can be induced within as little as four hours in THP-1 cells differentiated to a mature Mø phenotype. These findings indicate that smoking reduces Mø TLR3 expression directly, and not secondarily due to an effect on another lung cell type or on the lung microbiome.

### AMø of smokers show reduced CXCL10 production in response to poly(I:C) stimulation in vitro

**Figure 5:**
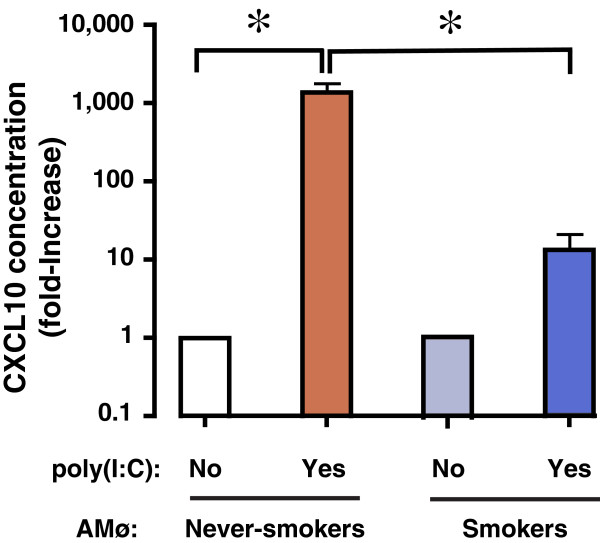
**Production of CXCL10 by AMø of smokers in response to poly(I:C) stimulation is impaired.** AMø from never-smokers (*n* = 4) (white and red columns) and smokers (*n* = 5) (light and dark blue columns) were cultured for 24 h in either complete medium alone or with poly(I:C). Supernatants were collected and CXCL10 protein was measured by Luminex assay; results are expressed as the fold-increase in CXCL10 over the unstimulated control condition; note the logarithmic scale of the vertical axis. Data are mean ± SEM. One-way ANOVA with Bonferroni’s Multiple Comparison post-hoc testing was used to compare differences among groups, *, *p* < 0.01.

Finally, we investigated whether decreased TLR3 expression on the AMø of smokers would affect CXCL10 production following poly(I:C) stimulation. These experiments were performed using AMø from subjects in the BAL cohort (Additional file 2: Table S2). CXCL10 concentrations were close to the level of detection in unstimulated cells from both smokers (*n* = 5) and never-smokers (*n* = 4) (Table 4) and did not differ significantly (Figure 5). Following poly(I:C) stimulation, AMø from never-smokers showed a 1000-fold increase in CXCL10 production, and differed significantly from the response of AMø from smokers, which showed only a 10-fold increase from unstimulated levels (Figure 5). These results illustrate a functional consequence of the difference in TLR3 expression between the two groups.

**Table 4:** Characteristics of BAL subjects used for in vitro stimulation studies

**Group**	**Smokers**	**Never-smokers**	***p *value**
Subjects, *n*	5	4	
Sex ratio, M/F	5/0	2/2	0.13
Age, years (SD)	60 (11)	39.5 (12.1)	0.06
Smoking, pack-years (SD)	47.4 (35.2)	0 (0)	0.02
Smoking status (Former/Current)	0/5	0/0	0.007
FEV_1_, % pred. (SD)	66.6 (30.6)	106.5 (12)	0.06
FEV1/FVC% (SD)	61.8 (19)	81.0 (7.5)	0.10
ICS use (yes/no)	3/2	0/4	0.10

*ICS* Inhaled Corticosteroid Use, *M* Male, *F* Female. Data are represented as mean (SD), ratio of current smokers to former smokers, or fraction of ICS users (yes/no). All FEV1 values are pre-bronchodilator. *p* values calculated using Mann Whitney test.

## Discussion

We demonstrate that smoking reduces expression by human lung Mø of TLR3, a receptor for microbial dsRNA and endogenous danger signals. Relative to AMø of never-smokers, AMø of current smokers showed reduced TLR3 mRNA and protein, and lower secretion of CXCL10 in response to the viral dsRNA analogue poly(I:C). Smoking did not significantly reduce AMø expression of other receptors for viral nucleic acids in the cytoplasm (RIG-I, MDA-5 or PKR) or endosome (TLR7, TLR8 or TLR9). In a separate cohort, TLR3 protein expression by total lung Mø was also reduced in active smokers, relative to former smokers. TLR3 protein expressed as the percentage of postive lung Mø did not correlate significantly in either cohort with a diagnosis of COPD, FEV1% predicted or total history of smoking expressed as pack-years. The direct nature of the smoking effect was shown in vitro using differentiated cells of the human Mø line THP-1. These findings identify a new mechanism by which smoking impairs anti-viral lung host defenses.

Our findings address the intersection of two highly prevalent clinical issues, host defense against respiratory viruses and smoking. Respiratory viruses are implicated, alone or as mixed infections, in most COPD exacerbations, including those of greatest severity [31,32]. Viral respiratory infections are also the leading cause of asthma exacerbations in adults and children [33,34], including those requiring hospitalization [35]. Although only some smokers develop COPD, those who continue to smoke sustain more frequent respiratory infections and faster lung function decline [36]. Smoking is also a key contributor to morbidity in asthma [37]. Asthmatic smokers experience worse symptom control, greater risk of hospitalization, and increased resistance to the therapeutic effects of inhaled corticosteroids [38-41]. Finally, even in individuals with normal spirometry, smoking is an independent risk factor for increased number and severity of respiratory infections [3]. Hence, these results provide another rationale to urge all smokers to quit smoking absolutely, regardless of pulmonary function or specific diagnosis.

Optimal defense against respiratory viruses requires coordination of multiple elements of innate and adaptive immunity [42], especially prompt production by epithelial cells of types I (alpha/beta) and III (lambda) IFNs [43-45]. Appropriate contributions by AMø are likely also important [42]. In response to viruses, AMø produce measurable amounts of type I IFNs [46] and of the type III IFN lambda 1 (IL-29) [47]. Human monocyte-derived Mø (MDMø) produce mRNA for type III IFNs in response to stimultation via TLR3 or TLR4 [48] or to viruses [47,49]. A specific anti-viral role of AMø has been confirmed experimentally by cellular depletion in two animal models [50,51], but in a murine model of respiratory syncytial virus pneumonia, AMø depletion reduced early antiviral responses, increased viral load, but had no effect on late outcomes [52]. Hence, the significance of the current findings may vary with the particular viral pathogen. Additional studies will be needed to determine whether the effect we found on TLR3 expression translates into defective responses by the AMø of smokers to intact respiratory viruses.

These results expand the known effects of cigarette smoking on AMø function, which are clearly complex. Two studies using Affymetrix Human Genome U133 Plus 2.0 GeneChips found AMø from human smokers to have distinctive, globally altered gene expression profiles relative to non-smokers [53,54]. Interestingly, TLR3 is not mentioned as a differentially expressed gene in either study. Disparity from our results likely relates to the difference in methodology; importantly, we showed very reduced TLR3 protein expression. The more pronounced reduction we found in TLR3 protein relative to mRNA implies the possibility of post-transcriptional smoking-induced downregulation, an important issue for future studies.

AMø of human smokers are often described as being “activated” [55-57], but both they and AMø from COPD patients show impaired bacterial uptake and killing [58,59]. Production of TNF-α and IL-6 by AMø of smokers (and COPD patients, which often mixed current and former smokers) equaled or exceeded that of non-smokers, but was strikingly less inhibitable by steroids [60,61]. Increased secretion of IL-8 and other neutrophil-attracting chemokines by AMø of human smokers has been shown in most [62,63] but not all [64] studies. Thus, smoking appears to make AMø less likely to respond efficiently to pathogens but more likely to enhance sustained inflammation.

TLR3 has been shown experimentally to be essential for optimal protection against some but not all viruses, even those with a dsRNA genome [17]. Moreover, under some circumstances TLR3 may be responsible for exaggerated inflammation [65], and anti-TLR3 blocking antibody is being developed for therapeutic use [66]. Importantly, TLR3 is a receptor not only for microbial dsRNA, but also for dsRNA from necrotic host cells [67], an example of an endogenous danger-associated molecular pattern. This finding assumes increased significance based on the recognition of increased apoptotic cell death in emphysema [68] and decreased clearance of apoptotic cells by AMø of smokers [69-71], a defect postulated to foster lung injury and emphysema progression [71,72]. Hence, the putative detrimental effect of reduced TLR3 expression by AMø of smokers on antiviral defenses might be partially offset by reduced responsiveness to necrosis-induced lung inflammation, an intriguing possibility that will require considerably greater investigation. Our finding that the percentage of TLR3-positive AMø was increased in former smokers when compared to active smokers agrees with the partial resolution of other smoking-induced AMø defects on smoking cessation [73]. These data are also congruent with the observation that smoking but not COPD decreases AMø expression of TLR2, but not of TLR4 [74]. The molecular mechanisms for down regulation by active smoking of TLR3 (this study) and TLR2 [74] remain unclear.

Our data partially differ from those of Koarai and colleagues, who recently reported increased TLR3 expression by AMø of smokers that correlated positively with smoking history and inversely with DLCO but not FEV1 % predicted [30]. Our results agree in finding no differences in AMø TLR3 expression among smokers based on the presence or absence of spirometrically-defined COPD. That study examined paraffin-fixed sections using a polyclonal anti-TLR3 antibody and immunohistochemistry, whereas we analyzed freshly isolated cells using the same monoclonal antibody in both of our cohorts. Hence, the disparity regarding TLR3 expression results might relate to antigen availability. Our studies differ in the cell type used for in vitro exposure to CSE (in that study MDMø), incubation time (24 hr vs. 4 hr in the current study) and CSE concentration (5% vs. a significant effect at 1.25% in the current study). These disparities make comparison difficult, as do multiple differences in design and endpoints of the experiments testing poly(I:C)-stimulated mediator production. One other study found that CSE reduced mRNA expression by MDMø of TLR8 and MDA5 and that MDMø of smokers had reduced mRNA expression of RIG-I and MDA-5, relative to MDMø of never-smokers [75]. That study does not mention TLR3 expression, which presumably was not altered in either case. Differences from our results likely reflect disparity both in cell type studied and possibly in methodology, as those results were obtained by Affimetrix assay.

The current findings are interesting in regard to two recent murine studies. Gaschler and colleagues found attenuated in vitro secretion of TNF-α, IL-6 and CCL5 in response to poly(I:C) by AMø from smoke-exposed mice, but did not find decreased TLR3 expression [76]. That disparity from our results may reflect a species difference. Kang and colleagues found that cigarette smoke selectively augmented airway and alveolar inflammation induced by viral MAMPs and influenza virus, as measured by induction of type I IFN, IFN-γ, IL-18 and IL-12/IL-23 p40 and PKR activation [77]. Further analysis using knockout mice indicated a role for both acute TLR3-dependent and chronic TLR3-independent pathways, as well as a pathway dependent on mitochondrial antiviral signaling protein (MAVS), IL-18Rα, IFN-γ and PKR [77]. That important study, however, did not measure TLR3 expression by AMø, and its findings likely reflect the interaction of multiple cell types in vivo.

Considerable evidence indicates the importance of CXCL10 in antiviral host defense but also in development or prevention of certain lung diseases [78,79]. CXCL10 is one of three chemokines, along with CXCL9 and CXCL11, that are highly induced by IFN-γ, as well as by types I and III IFNs [49,80]. Of note, however, CXCL10 production is also induced directly by infection of human AMø by rhinovirus, of murine AMø by RSV and of human MDMø by influenza or HIV [19,20,81-83], making this cytokine a suitable endpoint to test the functional importance of the observed TLR3 downregulation. A key action of CXCL10 is inflammatory cell recruitment, acting via CXCR3, which is found on Mø, CD8+ T cells, activated CD4+ T cells (especially T_H_1 cells), NK cells and plasmacytoid dendritic cells [84]. CXCR3 is essential for recruitment of antigen-specific CD4+ T cells to the lungs in a murine model of parainfluenza pneumonia [85]. In a model of Coxsackievirus infection, CXCL10 was shown to limit viral replication via NK cell recruitment [24].

Markedly reduced induction of CXCL10 in AMø from smokers is of interest in the context of other recent human studies. CXCL10 is one of several M1-associated genes down-regulated in AMø of healthy smokers and even more so, in smokers with COPD, relative to healthy never-smokers [54]. Although significantly increased CXCL10 concentrations have been found in the sputum of COPD patients, when compared with nonsmokers but not with smokers without obstruction [86], the origin of that chemokine is unclear, as it is also produced by epithelial cells that significantly outnumber Mø in the airways. Both by showing the role of reduced TLR3 expression and by studying AMø, we extend a study of gene expression by MDMø, which found reduced expression of CXCL10 at baseline in smokers relative to never-smokers, and also that CSE reduced IFN-γ-induced CXCL10 production by MDMø of never-smokers [75].

Our study has several limitations. One is the absence of never-smokers from our surgical cohort, who were recruited before clinically-indicated resections. Another is the predominance in our BAL cohort of female never-smokers (11 of 13 subjects) versus a predominance of male current smokers with preserved lung function (eight of nine subjects) and COPD patients (11 of 11 subjects). Concerns that differences in sex or age could confound our BAL results should be reduced by the congruent effect of smoking in our surgical cohort, which comprised 40% female subjects and which showed no significant effect of age on TLR3 expression by lung Mø. Moreover, at equivalent levels of tobacco exposure, women appear to be at greater risk of lung function impairment {reviewed in [87]}, the opposite of what would be expected if the current finding were a male-specific effect. Although we cannot formally exclude the possibility that differences in subject age explain the reduced expression of CXCL10 in response to TLR3, we consider that possibility unlikely, as serum levels of CXCL10 increase with age [88,89]. Other limitations are the absence of DLCO measurements, which precludes comparison of our results with those of Koarai and colleagues [30] on this point, and that because post-bronchodilator FEV1 values were not available on all subjects, we have presented and analyzed exclusively pre-bronchodilator FEV1 values.

## Conclusions

Active smoking reduces expression of TLR3 by human lung Mø, assayed both as AMø harvested by BAL and as a mixture of AMø and interstitial lung Mø from surgical tissue. This effect occurs via both down-regulation of mRNA transcripts, which can be induced in vitro within four hours, and from a more pronounced effect on TLR3 protein expression, which appears to be at least partially reversible on smoking cessation. Reduction in lung Mø TLR3 expression may be one mechanism contributing to the increased incidence of viral respiratory infections in smokers and to viral induction of acute exacerbations of COPD. Defining the effect of smoking on the phenotype of human AMø and other lung cell types is a crucial step in the translation of basic science into therapies.

### Endnotes

Subjects were recruited via observational studies registered with ClinicalTrials.gov as NCT00281190, NCT00281203 and NCT00281229. These data were presented in part at the International Scientific Conference of the American Thoracic Society, May 17, 2010 in New Orleans, LA, and have been published in abstract form *Am J Respir Crit Care Med* 2010; 181: A3875.

Supported by R01 HL082480 (JLC, FJM), R01 HL056309 (JLC) and U01 HL098961 (JMB, JLC) from the USPHS; a Career Development Award (CMF) and a Merit Review Award (CMF) and a Research Enhancement Award Program (SWC, JMB, JLC) from the Biomedical Laboratory Research & Development Service, Department of Veterans Affairs. These investigations were also supported in part by the Tissue Procurement Core of the University of Michigan Comprehensive Cancer Center, Grant P30 CA46952, and by the Lung Tissue Research Consortium (Clinical Centers), Grant N01 HR046162.

Current e-mail addresses: James M. Beck, M.D.: http://james.beck@denver.edu.

## Abbreviations

AMø: Alveolar macrophage(s); BAL: Bronchoalveolar lavage; COPD: Chronic obstructive pulmonary disease; CSE: Cigarette smoke extract; Ds: Double-stranded; FEV1: Forced expiratory volume in 1 second; FVC: Forced vital capacity; GAPDH: Glyceraldehyde-3-phosphate dehydrogenase; Mø: Macrophage(s); MDA-5: Melanoma differentiation-associated gene 5; MDMø: Monocyte-derived macrophage(s); NK: Natural killer; PKR: Double-stranded RNA-dependent protein kinase; PMA: Phorbol myristate acetate; poly(I:C): Polyinosinic acid:cytidylic acid; RIG-I: Retinoic acid-inducible gene I; TLR3: Toll-like receptor 3.

## Competing interests

Jill C Todt, Jeanette P Brown, Joanne Sonstein, Theresa M Ames, Alexandra McCubbrey and Stephen W Chensue have no competing interests to declare. Christine M Freeman, Fernando J Martinez, James M Beck and Jeffrey L Curtis were supported by research grants as outlined in the Endnotes, but have no other competing interests to declare.

## Authors’ contributions

JCT: Designed and performed experiments, analyzed data, produced graphs and tables, wrote the initial draft of manuscript, reviewed and approved final manuscript; CMF: designed and performed experiments, analyzed data and produced graphs and tables, wrote the initial draft of the revised manuscript, reviewed and approved final manuscript; JPB designed and performed experiments, analyzed data and produced graphs and tables, reviewed and approved final manuscript; JS: performed and analyzed flow cytometry experiments, reviewed and approved final manuscript; TMA: performed and photographed immunocytochemistry experiments, participated in performance of other experiments, reviewed and approved final manuscript; ALM: designed and performed experiments, reviewed and approved final manuscript; FJM: secured research funding, participated in study design, analyzed data, participated in manuscript generation, reviewed and approved final manuscript; SWC: performed dissection of surgical lung specimens, reviewed and approved final manuscript; JMB: secured research funding, participated in study design, analyzed data, participated in manuscript generation, reviewed and approved final manuscript; JLC: secured research funding, oversaw study design, performed all subject-related activities including bronchoscopies, analyzed data, generated the final manuscript, and takes responsibility for the scientific integrity of the overall project.

## Supplementary Material

Additional file 1: Table S1 Summary of all BAL subjects demographics, smoking histories, spirometry & current smoking status. ICS, inhaled corticosteroid use; FC, flow cytometry; IHC, immunohistochemistry. Summarized data are shown in bold and are represented as mean (SD), ratio of current smokers to former smokers, or fraction of ICS users (yes/no). All FEV1 values are pre-bronchodilator. Click here for file

Additional file 2: Table S2 Summary of all lung tissue subject demographics, smoking histories, spirometry & current smoking status. ICS, inhaled corticosteroid use. M, Male; F, Female. Summarized data are shown in bold and are represented as mean (SD), ratio of current smokers to former smokers, or fraction of ICS users (yes/no). All FEV1 values are pre-bronchodilator. Click here for file

Additional file 3: Figure S1 AMø of current smokers show no reduction in mRNA expression of cytoplasmic dsRNA receptors, relative to AMø of never-smokers. RNA from AMø was isolated, depleted of contaminating genomic DNA, reverse-transcribed and analyzed by quantitative real-time RT-PCR using Taqman chemistry and specific primer-probe sets, normalized to GAPDH transcripts. Data are expressed on the horizontal axis as mean ± SEM for relative quantity (dRn), calculated in comparison to a single never-smoker who was arbitrarily designated the reference sample. Never-smokers (*n* = 6), red bars; smokers (*n* = 11), blue bars. The Mann–Whitney test was used to calculate statistical significance. Click here for file

Additional file 4: Figure S2 Representative flow cytometry results. AMø were permeabilized, stained for expression of TLR3 (left-hand panels), TLR 7 (middle panel) and or TLR9 (right-hand panels), and analyzed by flow cytometry, gating on AMø (CD45+, high side scatter cells). A,B; specific staining (red line), isotype control staining (blue line). A, never-smoker, B, smoker. Click here for file

Additional file 5: Figure S3 Lack of correlation of spirometry or total smoking history with lung AMø TLR3 expresssion in surgical cohort. Total lung Mø were harvested from excess lung tissue removed surgically for clinical indications as decribed in the legend to Figure 3. Lung Mø were permeabilized, stained for TLR3 expression and analyzed by flow cytometry, gating on CD45+, high side-scatter cells. Data are shown as the percentage of TLR3-positive lung Mø on the vertical axis versus A, FEV1 % predicted; B, smoking history in pack-years. In panel A, circles represent current smokers and “x” respresents former smokers. In panel B, all subjects are shown as inverted triangles, regardless of smoking status (active vs. former); in both panels, *n* = 25. Click here for file

## References

[B1] Johnston SL Overview of virus-induced airway disease Proc Am Thorac Soc 2005 2 150 156 10.1513/pats.200502-018AW 16113484

[B2] Proud D Chow CW Role of viral infections in asthma and chronic obstructive pulmonary disease Am J Respir Cell Mol Biol 2006 35 513 518 10.1165/rcmb.2006-0199TR 16778148

[B3] Arcavi L Benowitz NL Cigarette smoking and infection Arch Intern Med 2004 164 2206 2216 10.1001/archinte.164.20.2206 15534156

[B4] Aronson MD Weiss ST Ben RL Komaroff AL Association between cigarette smoking and acute respiratory tract illness in young adults JAMA 1982 248 181 183 10.1001/jama.1982.03330020025023 7087108

[B5] Bensenor IM Cook NR Lee IM Chown MJ Hennekens CH Buring JE Manson JE Active and passive smoking and risk of colds in women Ann Epidemiol 2001 11 225 231 10.1016/S1047-2797(00)00214-3 11306340

[B6] Marcy TW Merrill WW Cigarette smoking and respiratory tract infection Clin Chest Med 1987 8 381 391 3311582

[B7] Sopori M Effects of cigarette smoke on the immune system Nat Rev Immunol 2002 2 372 377 10.1038/nri803 12033743

[B8] Mehta H Nazzal K Sadikot RT Cigarette smoking and innate immunity Inflamm Res 2008 57 497 503 10.1007/s00011-008-8078-6 19109742

[B9] Matsunaga K Klein TW Friedman H Yamamoto Y Involvement of nicotinic acetylcholine receptors in suppression of antimicrobial activity and cytokine responses of alveolar macrophages to Legionella pneumophila infection by nicotine J Immunol 2001 167 6518 6524 11714820 10.4049/jimmunol.167.11.6518

[B10] Ouyang Y Virasch N Hao P Aubrey MT Mukerjee N Bierer BE Freed BM Suppression of human IL-1beta, IL-2, IFN-gamma, and TNF-alpha production by cigarette smoke extracts J Allergy Clin Immunol 2000 106 280 287 10.1067/mai.2000.107751 10932071

[B11] Wewers MD Diaz PT Wewers ME Lowe MP Nagaraja HN Clanton TL Cigarette smoking in HIV infection induces a suppressive inflammatory environment in the lung Am J Respir Crit Care Med 1998 158 1543 1549 9817706 10.1164/ajrccm.158.5.9802035

[B12] Chen H Cowan MJ Hasday JD Vogel SN Medvedev AE Tobacco smoking inhibits expression of proinflammatory cytokines and activation of IL-1R-associated kinase, p38, and NF-kappaB in alveolar macrophages stimulated with TLR2 and TLR4 agonists J Immunol 2007 179 6097 6106 17947684 10.4049/jimmunol.179.9.6097

[B13] Birrell MA Wong S Catley MC Belvisi MG Impact of tobacco-smoke on key signaling pathways in the innate immune response in lung macrophages J Cell Physiol 2008 214 27 37 10.1002/jcp.21158 17541958

[B14] Edwards K Braun KM Evans G Sureka AO Fan S Mainstream and sidestream cigarette smoke condensates suppress macrophage responsiveness to interferon gamma Hum Exp Toxicol 1999 18 233 240 10.1191/096032799678839978 10333308

[B15] Matsumoto M Funami K Tanabe M Oshiumi H Shingai M Seto Y Yamamoto A Seya T Subcellular localization of Toll-like receptor 3 in human dendritic cells J Immunol 2003 171 3154 3162 12960343 10.4049/jimmunol.171.6.3154

[B16] Orinska Z Bulanova E Budagian V Metz M Maurer M Bulfone-Paus S TLR3-induced activation of mast cells modulates CD8+ T-cell recruitment Blood 2005 106 978 987 10.1182/blood-2004-07-2656 15840693

[B17] Meylan E Tschopp J Toll-like receptors and RNA helicases: two parallel ways to trigger antiviral responses Mol Cell 2006 22 561 569 10.1016/j.molcel.2006.05.012 16762830

[B18] Matsukura S Kokubu F Kurokawa M Kawaguchi M Ieki K Kuga H Odaka M Suzuki S Watanabe S Homma T Role of RIG-I, MDA-5, and PKR on the expression of inflammatory chemokines induced by synthetic dsRNA in airway epithelial cells Int Arch Allergy Immunol 2007 143 Suppl 1 80 83 17541283 10.1159/000101411

[B19] Hui KP Lee SM Cheung CY Ng IH Poon LL Guan Y Ip NY Lau AS Peiris JS Induction of proinflammatory cytokines in primary human macrophages by influenza A virus (H5N1) is selectively regulated by IFN regulatory factor 3 and p38 MAPK J Immunol 2009 182 1088 1098 19124752 10.4049/jimmunol.182.2.1088

[B20] Dhillon N Zhu X Peng F Yao H Williams R Qiu J Callen S Ladner AO Buch S Molecular mechanism(s) involved in the synergistic induction of CXCL10 by human immunodeficiency virus type 1 Tat and interferon-gamma in macrophages J Neurovirol 2008 14 196 204 10.1080/13550280801993648 18569454 PMC2715278

[B21] Livengood AJ Wu CC Carson DA Opposing roles of RNA receptors TLR3 and RIG-I in the inflammatory response to double-stranded RNA in a Kaposi’s sarcoma cell line Cell Immunol 2007 249 55 62 10.1016/j.cellimm.2007.11.004 18155685 PMC2262282

[B22] Michalec L Choudhury BK Postlethwait E Wild JS Alam R Lett-Brown M Sur S CCL7 and CXCL10 orchestrate oxidative stress-induced neutrophilic lung inflammation J Immunol 2002 168 846 852 11777981 10.4049/jimmunol.168.2.846

[B23] Agostini C Calabrese F Poletti V Marcer G Facco M Miorin M Cabrelle A Baesso I Zambello R Trentin L Semenzato G CXCR3/CXCL10 interactions in the development of hypersensitivity pneumonitis Respir Res 2005 6 20 10.1186/1465-9921-6-20 15725351 PMC554979

[B24] Yuan J Liu Z Lim T Zhang H He J Walker E Shier C Wang Y Su Y Sall A CXCL10 inhibits viral replication through recruitment of natural killer cells in coxsackievirus B3-induced myocarditis Circ Res 2009 104 628 638 10.1161/CIRCRESAHA.108.192179 19168435

[B25] Cole AM Ganz T Liese AM Burdick MD Liu L Strieter RM Cutting edge: IFN-inducible ELR- CXC chemokines display defensin-like antimicrobial activity J Immunol 2001 167 623 627 11441062 10.4049/jimmunol.167.2.623

[B26] Vasquez RE Xin L Soong L Effects of CXCL10 on dendritic cell and CD4+ T-cell functions during Leishmania amazonensis infection Infect Immun 2008 76 161 169 10.1128/IAI.00825-07 17998308 PMC2223631

[B27] Freeman CM Curtis JL Chensue SW CC chemokine receptor 5 and CXC chemokine receptor 6 expression by lung CD8+ cells correlates with chronic obstructive pulmonary disease severity Am J Pathol 2007 171 767 776 10.2353/ajpath.2007.061177 17640964 PMC1959492

[B28] Freeman CM Han MK Martinez FJ Murray S Liu LX Chensue SW Polak TJ Sonstein J Todt JC Ames TM Cytotoxic potential of lung CD8(+) T cells increases with chronic obstructive pulmonary disease severity and with in vitro stimulation by IL-18 or IL-15 J Immunol 2010 184 6504 6513 10.4049/jimmunol.1000006 20427767 PMC4098931

[B29] McCubbrey AL Sonstein J Ames TM Freeman CM Curtis JL Glucocorticoids relieve collectin-driven suppression of apoptotic cell uptake in murine alveolar macrophages through downregulation of SIRPalpha J Immunol 2012 189 112 119 10.4049/jimmunol.1200984 22615206 PMC3381851

[B30] Koarai A Yanagisawa S Sugiura H Ichikawa T Akamatsu K Hirano T Nakanishi M Matsunaga K Minakata Y Ichinose M Cigarette smoke augments the expression and responses of toll-like receptor 3 in human macrophages Respirology 2012 17 1018 1025 10.1111/j.1440-1843.2012.02198.x 22591330

[B31] Seemungal T Harper-Owen R Bhowmic A Moric I Sanderson G Message S MacCallum P Meade T Jeffries D Johnston S Wedzicha J Respiratory viruses, symptoms, and inflammatory markers in acute exacerbations and stable chronic obstructive pulmonary disease Am J Respir Crit Care Med 2001 164 1618 1623 11719299 10.1164/ajrccm.164.9.2105011

[B32] Papi A Bellettato C Braccioni F Romagnoli M Casolari P Caramori G Fabbri L Johnston S Infections and airway inflammation in chronic obstructive pulmonary disease severe exacerbations Am J Respir Crit Care Med 2006 173 1114 1121 10.1164/rccm.200506-859OC 16484677

[B33] Nicholson KG Kent J Ireland DC Respiratory viruses and exacerbations of asthma in adults BMJ 1993 307 982 986 10.1136/bmj.307.6910.982 8241910 PMC1679193

[B34] Johnston SL Pattemore PK Sanderson G Smith S Lampe F Josephs L Symington P O’Toole S Myint SH Tyrrell DA Community study of role of viral infections in exacerbations of asthma in 9–11 year old children BMJ 1995 310 1225 1229 10.1136/bmj.310.6989.1225 7767192 PMC2549614

[B35] Johnston SL Pattemore PK Sanderson G Smith S Campbell MJ Josephs LK Cunningham A Robinson BS Myint SH Ward ME The relationship between upper respiratory infections and hospital admissions for asthma: a time-trend analysis Am J Respir Crit Care Med 1996 154 654 660 8810601 10.1164/ajrccm.154.3.8810601

[B36] Kanner RE Anthonisen NR Connett JE Lower respiratory illnesses promote FEV(1) decline in current smokers but not ex-smokers with mild chronic obstructive pulmonary disease: results from the lung health study Am J Respir Crit Care Med 2001 164 358 364 11500333 10.1164/ajrccm.164.3.2010017

[B37] Thomson NC Chaudhuri R Asthma in smokers: challenges and opportunities Curr Opin Pulm Med 2009 15 39 45 10.1097/MCP.0b013e32831da894 19077704

[B38] Eisner MD Iribarren C The influence of cigarette smoking on adult asthma outcomes Nicotine Tob Res 2007 9 53 56 10.1080/14622200601078293 17365736

[B39] Sippel JM Pedula KL Vollmer WM Buist AS Osborne ML Associations of smoking with hospital-based care and quality of life in patients with obstructive airway disease Chest 1999 115 691 696 10.1378/chest.115.3.691 10084477

[B40] Siroux V Pin I Oryszczyn MP LeMoual N Kauffmann F Relationships of active smoking to asthma and asthma severity in the EGEA study. Epidemiological study on the Genetics and Environment of Asthma Eur Respir J 2000 15 470 477 10.1034/j.1399-3003.2000.15.08.x 10759439

[B41] Lazarus SC Chinchilli VM Rollings NJ Boushey HA Cherniack R Craig TJ Deykin A DiMango E Fish JE Ford JG Smoking affects response to inhaled corticosteroids or leukotriene receptor antagonists in asthma Am J Respir Crit Care Med 2007 175 783 790 10.1164/rccm.200511-1746OC 17204725 PMC1899291

[B42] Kohlmeier JE Woodland DL Immunity to respiratory viruses Annu Rev Immunol 2009 27 61 82 10.1146/annurev.immunol.021908.132625 18954284

[B43] Khaitov MR Laza-Stanca V Edwards MR Walton RP Rohde G Contoli M Papi A Stanciu LA Kotenko SV Johnston SL Respiratory virus induction of alpha-, beta- and lambda-interferons in bronchial epithelial cells and peripheral blood mononuclear cells Allergy 2009 64 375 386 10.1111/j.1398-9995.2008.01826.x 19175599

[B44] Contoli M Message SD Laza-Stanca V Edwards MR Wark PA Bartlett NW Kebadze T Mallia P Stanciu LA Parker HL Role of deficient type III interferon-lambda production in asthma exacerbations Nat Med 2006 12 1023 1026 10.1038/nm1462 16906156

[B45] Cakebread JA Xu Y Grainge C Kehagia V Howarth PH Holgate ST Davies DE Exogenous IFN-beta has antiviral and anti-inflammatory properties in primary bronchial epithelial cells from asthmatic subjects exposed to rhinovirus J Allergy Clin Immunol 2011 127 1148 1154 e1149 10.1016/j.jaci.2011.01.023 21329968

[B46] Laza-Stanca V Stanciu LA Message SD Edwards MR Gern JE Johnston SL Rhinovirus replication in human macrophages induces NF-kappaB-dependent tumor necrosis factor alpha production J Virol 2006 80 8248 8258 10.1128/JVI.00162-06 16873280 PMC1563804

[B47] Wang J Oberley-Deegan R Wang S Nikrad M Funk CJ Hartshorn KL Mason RJ Differentiated human alveolar type II cells secrete antiviral IL-29 (IFN-lambda 1) in response to influenza A infection J Immunol 2009 182 1296 1304 19155475 10.4049/jimmunol.182.3.1296 PMC4041086

[B48] Siren J Pirhonen J Julkunen I Matikainen S IFN-alpha regulates TLR-dependent gene expression of IFN-alpha, IFN-beta, IL-28, and IL-29 J Immunol 2005 174 1932 1937 15699120 10.4049/jimmunol.174.4.1932

[B49] Sheppard P Kindsvogel W Xu W Henderson K Schlutsmeyer S Whitmore TE Kuestner R Garrigues U Birks C Roraback J IL-28, IL-29 and their class II cytokine receptor IL-28R Nat Immunol 2003 4 63 68 10.1038/ni873 12469119

[B50] Kim HM Lee YW Lee KJ Kim HS Cho SW Van Rooijen N Guan Y Seo SH Alveolar macrophages are indispensable for controlling influenza viruses in lungs of pigs J Virol 2008 82 4265 4274 10.1128/JVI.02602-07 18287245 PMC2293066

[B51] Kumagai Y Takeuchi O Kato H Kumar H Matsui K Morii E Aozasa K Kawai T Akira S Alveolar macrophages are the primary interferon-alpha producer in pulmonary infection with RNA viruses Immunity 2007 27 240 252 10.1016/j.immuni.2007.07.013 17723216

[B52] Pribul PK Harker J Wang B Wang H Tregoning JS Schwarze J Openshaw PJ Alveolar macrophages are a major determinant of early responses to viral lung infection but do not influence subsequent disease development J Virol 2008 82 4441 4448 10.1128/JVI.02541-07 18287232 PMC2293049

[B53] Woodruff PG Koth LL Yang YH Rodriguez MW Favoreto S Dolganov GM Paquet AC Erle DJ A distinctive alveolar macrophage activation state induced by cigarette smoking Am J Respir Crit Care Med 2005 172 1383 1392 10.1164/rccm.200505-686OC 16166618 PMC2718436

[B54] Shaykhiev R Krause A Salit J Strulovici-Barel Y Harvey BG O’Connor TP Crystal RG Smoking-dependent reprogramming of alveolar macrophage polarization: implication for pathogenesis of chronic obstructive pulmonary disease J Immunol 2009 183 2867 2883 10.4049/jimmunol.0900473 19635926 PMC2873685

[B55] Hunninghake GW Gadek JE Kawanami O Ferrans VJ Crysta l RG Inflammatory and immune processes in the human lung in health and disease. Evaluation by bronchoalveolar lavage Am J Pathol 1979 97 149 206 495693 PMC2042387

[B56] Richards SW Peterson PK Verbrugh HA Nelson RD Hammerschmidt DE Hoidal JR Chemotactic and phagocytic responses of human alveolar macrophages to activated complement components Infect Immun 1984 43 775 778 6693176 10.1128/iai.43.2.775-778.1984 PMC264372

[B57] Holt PG Immune and inflammatory function in cigarette smokers Thorax 1987 42 241 249 10.1136/thx.42.4.241 3303428 PMC460693

[B58] Marti-Lliteras P Regueiro V Morey P Hood DW Saus C Sauleda J Agusti AG Bengoechea JA Garmendia J Nontypeable Haemophilus influenzae clearance by alveolar macrophages is impaired by exposure to cigarette smoke Infect Immun 2009 77 4232 4242 10.1128/IAI.00305-09 19620348 PMC2747942

[B59] King TE Jr Savici D Campbell PA Phagocytosis and killing of Listeria monocytogenes by alveolar macrophages: smokers versus nonsmokers J Infect Dis 1988 158 1309 1316 10.1093/infdis/158.6.1309 3143765

[B60] Cosio BG Tsaprouni L Ito K Jazrawi E Adcock IM Barnes PJ Theophylline restores histone deacetylase activity and steroid responses in COPD macrophages J Exp Med 2004 200 689 695 10.1084/jem.20040416 15337792 PMC2212744

[B61] Cosio MG Saetta M Agusti A Immunologic aspects of chronic obstructive pulmonary disease N Engl J Med 2009 360 2445 2454 10.1056/NEJMra0804752 19494220

[B62] McCrea KA Ensor JE Nall K Bleecker ER Hasday JD Altered cytokine regulation in the lungs of cigarette smokers Am J Respir Crit Care Med 1994 150 696 703 8087340 10.1164/ajrccm.150.3.8087340

[B63] Morrison D Strieter RM Donnelly SC Burdick MD Kunkel SL MacNee W Neutrophil chemokines in bronchoalveolar lavage fluid and leukocyte-conditioned medium from nonsmokers and smokers Eur Respir J 1998 12 1067 1072 10.1183/09031936.98.12051067 9863998

[B64] Ohta T Yamashita N Maruyama M Sugiyama E Kobayashi M Cigarette smoking decreases interleukin-8 secretion by human alveolar macrophages Respir Med 1998 92 922 927 10.1016/S0954-6111(98)90191-3 10070565

[B65] Hutchens M Luker KE Sottile P Sonstein J Lukacs NW Nunez G Curtis JL Luker GD TLR3 increases disease morbidity and mortality from vaccinia infection J Immunol 2008 180 483 491 18097050 10.4049/jimmunol.180.1.483 PMC4470388

[B66] Bunting RA Duffy KE Lamb RJ San Mateo LR Smalley K Raymond H Liu X Petley T Fisher J Beck H Novel antagonist antibody to TLR3 blocks poly(I:C)-induced inflammation in vivo and in vitro Cell Immunol 2011 267 9 16 10.1016/j.cellimm.2010.10.008 21092943

[B67] Cavassani KA Ishii M Wen H Schaller MA Lincoln PM Lukacs NW Hogaboam CM Kunkel SL TLR3 is an endogenous sensor of tissue necrosis during acute inflammatory events J Exp Med 2008 205 2609 2621 10.1084/jem.20081370 18838547 PMC2571935

[B68] Henson PM Tuder RM Apoptosis in the lung: induction, clearance and detection Am J Physiol Lung Cell Mol Physiol 2008 294 L601 L611 10.1152/ajplung.00320.2007 18178675

[B69] Hodge S Hodge G Brozyna S Jersmann H Holmes M Reynolds PN Azithromycin increases phagocytosis of apoptotic bronchial epithelial cells by alveolar macrophages Eur Respir J 2006 28 486 495 10.1183/09031936.06.00001506 16737992

[B70] Hodge S Hodge G Scicchitano R Reynolds PN Holmes M Alveolar macrophages from subjects with chronic obstructive pulmonary disease are deficient in their ability to phagocytose apoptotic airway epithelial cells Immunol Cell Biol 2003 81 289 296 10.1046/j.1440-1711.2003.t01-1-01170.x 12848850

[B71] Richens TR Linderman DJ Horstmann SA Lambert C Xiao YQ Keith RL Boe DM Morimoto K Bowler RP Day BJ Cigarette smoke impairs clearance of apoptotic cells through oxidant-dependent activation of RhoA Am J Respir Crit Care Med 2009 179 1011 1021 10.1164/rccm.200807-1148OC 19264974 PMC2689911

[B72] Vandivier RW Henson PM Douglas IS Burying the dead: the impact of failed apoptotic cell removal (efferocytosis) on chronic inflammatory lung disease Chest 2006 129 1673 1682 10.1378/chest.129.6.1673 16778289

[B73] Hodge S Hodge G Ahern J Jersmann H Holmes M Reynolds PN Smoking alters alveolar macrophage recognition and phagocytic ability: implications in chronic obstructive pulmonary disease Am J Respir Cell Mol Biol 2007 37 748 755 10.1165/rcmb.2007-0025OC 17630319

[B74] Drömann D Goldmann T Tiedje T Zabel P Dalhoff K Schaaf B Toll-like receptor 2 expression is decreased on alveolar macrophages in cigarette smokers and COPD patients Respir Res 2005 6 68 10.1186/1465-9921-6-68 16004610 PMC1187924

[B75] Doyle I Ratcliffe M Walding A Vanden Bon E Dymond M Tomlinson W Tilley D Shelton P Dougall I Differential gene expression analysis in human monocyte-derived macrophages: impact of cigarette smoke on host defence Mol Immunol 2010 47 1058 1065 10.1016/j.molimm.2009.11.008 20022114

[B76] Gaschler GJ Zavitz CC Bauer CM Skrtic M Lindahl M Robbins CS Chen B Stampfli MR Cigarette smoke exposure attenuates cytokine production by mouse alveolar macrophages Am J Respir Cell Mol Biol 2008 38 218 226 17872497 10.1165/rcmb.2007-0053OC

[B77] Kang MJ Lee CG Lee JY Dela Cruz CS Chen ZJ Enelow R Elias JA Cigarette smoke selectively enhances viral PAMP- and virus-induced pulmonary innate immune and remodeling responses in mice J Clin Invest 2008 118 2771 2784 18654661 10.1172/JCI32709 PMC2483678

[B78] Bonecchi R Galliera E Borroni EM Corsi MM Locati M Mantovani A Chemokines and chemokine receptors: an overview Front Biosci 2009 14 540 551 19273084 10.2741/3261

[B79] Jiang D Liang J Campanella GS Guo R Yu S Xie T Liu N Jung Y Homer R Meltzer EB Inhibition of pulmonary fibrosis in mice by CXCL10 requires glycosaminoglycan binding and syndecan-4 J Clin Invest 2010 120 2049 2057 10.1172/JCI38644 20484822 PMC2877927

[B80] Pekarek V Srinivas S Eskdale J Gallagher G Interferon lambda-1 (IFN-lambda1/IL-29) induces ELR(−) CXC chemokine mRNA in human peripheral blood mononuclear cells, in an IFN-gamma-independent manner Genes Immun 2007 8 177 180 10.1038/sj.gene.6364372 17252004

[B81] Korpi-Steiner NL Bates ME Lee WM Hall DJ Bertics PJ Human rhinovirus induces robust IP-10 release by monocytic cells, which is independent of viral replication but linked to type I interferon receptor ligation and STAT1 activation J Leukoc Biol 2006 80 1364 1374 10.1189/jlb.0606412 17020930

[B82] Miller AL Bowlin TL Lukacs NW Respiratory syncytial virus-induced chemokine production: linking viral replication to chemokine production in vitro and in vivo J Infect Dis 2004 189 1419 1430 10.1086/382958 15073679

[B83] Proost P Vynckier AK Mahieu F Put W Grillet B Struyf S Wuyts A Opdenakker G Van Damme J Microbial Toll-like receptor ligands differentially regulate CXCL10/IP-10 expression in fibroblasts and mononuclear leukocytes in synergy with IFN-gamma and provide a mechanism for enhanced synovial chemokine levels in septic arthritis Eur J Immunol 2003 33 3146 3153 10.1002/eji.200324136 14579283

[B84] D’Ambrosio D Mariani M Panina-Bordignon P Sinigaglia F Chemokines and their receptors guiding T lymphocyte recruitment in lung inflammation Am J Respir Crit Care Med 2001 164 1266 1275 11673221 10.1164/ajrccm.164.7.2103011

[B85] Kohlmeier JE Cookenham T Miller SC Roberts AD Christensen JP Thomsen AR Woodland DL CXCR3 directs antigen-specific effector CD4+ T cell migration to the lung during parainfluenza virus infection J Immunol 2009 183 4378 4384 10.4049/jimmunol.0902022 19734208 PMC2757292

[B86] Costa C Rufino R Traves SL Lapa ESJR Barnes PJ Donnelly LE CXCR3 and CCR5 chemokines in induced sputum from patients with COPD Chest 2008 133 26 33 10.1378/chest.07-0393 17925429

[B87] Han MK Postma D Mannino DM Giardino ND Buist S Curtis JL Martinez FJ Gender and chronic obstructive pulmonary disease: why it matters Am J Respir Crit Care Med 2007 176 1179 1184 10.1164/rccm.200704-553CC 17673696 PMC2720110

[B88] Antonelli A Rotondi M Fallahi P Ferrari SM Paolicchi A Romagnani P Serio M Ferrannini E Increase of CXC chemokine CXCL10 and CC chemokine CCL2 serum levels in normal ageing Cytokine 2006 34 32 38 10.1016/j.cyto.2006.03.012 16697212

[B89] Antonelli A Rotondi M Fallahi P Romagnani P Ferrari SM Ferrannini E Serio M Age-dependent changes in CXC chemokine ligand 10 serum levels in euthyroid subjects J Interferon Cytokine Res 2005 25 547 552 10.1089/jir.2005.25.547 16181055

