# Supplementary material for: Smoking decreases the response of human lung macrophages to double-stranded RNA by reducing TLR3 expression
Source: Respir Res. 2013 Mar 9;14(1):33. doi: 10.1186/1465-9921-14-33 (PMC3599854; doi:10.1186/1465-9921-14-33)
Supplement: Additional file 1: Table S1 — Summary of all BAL subjects demographics, smoking histories, spirometry & current smoking status. ICS, inhaled corticosteroid use; FC, flow cytometry; IHC, immunohistochemistry. Summarized data are shown in bold and are represented as mean (SD), ratio of current smokers to former smokers, or fraction of ICS users (yes/no). All FEV1 values are pre-bronchodilator. [file 1465-9921-14-33-S1.doc]

**Supplemental Table S1. Summary of all BAL subjects demographics, smoking histories, spirometry & current smoking status**

|  | **Age** | **Sex** | **Smoking history (pack-years)** | **FEV_1_% predicted** | **FEV_1_/FVC, %** | **Smoking Status** | **Solitary nodule (Yes/No)** | **ICS (Yes/No)** | **Experiment used** |
| --- | --- | --- | --- | --- | --- | --- | --- | --- | --- |
| **Never smokers** | |  |  |  |  |  |  |  |  |
|  | 23 | F | 0 | 106 | 91 | Never | No | No | RNA |
|  | 41 | M | 0 | 111 | 83 | Never | No | No | RNA, IV |
|  | 26 | M | 0 | 89 | 79 | Never | No | No | RNA, IV, FC |
|  | 54 | F | 0 | 95 | 87 | Never | No | No | RNA, FC |
|  | 36 | F | 0 | 110 | 90 | Never | No | No | RNA, IV, FC |
|  | 46 | F | 0 | 100 | 91 | Never | No | No | RNA, FC, IHC |
|  | 55 | F | 0 | 116 | 72 | Never | No | No | IV |
|  | 21 | F | 0 | 116 | 86 | Never | No | No | FC, IHC |
|  | 30 | F | 0 | 84 | 88 | Never | No | No | FC |
|  | 58 | F | 0 | 91 | 78 | Never | No | No | FC |
|  | 39 | F | 0 | 107 | 87 | Never | No | No | FC |
|  | 54 | F | 0 | 107 | 83 | Never | No | No | FC |
|  | 58 | F | 0 | 108 | 74 | Never | No | No | FC, IHC |
| **Summary** | **41.6 (13.6)** | **2M/11F** | **0 (0)** | **103.1 (10.4)** | **83.8 (6.3)** | **0/13** | **0/13** | **0/13** |  |

**Supplemental Table S1. (continued)**

|  | **Age** | **Sex** | **Smoking history (pack-years)** | **FEV_1_% predicted** | **FEV_1_/FVC, %** | **Smoking Status** | **Solitary nodule (Yes/No)** | **ICS (Yes/No)** | **Experiment used** |
| --- | --- | --- | --- | --- | --- | --- | --- | --- | --- |
| **Smoking Controls** | |  |  |  |  |  |  |  |  |
|  | 54 | M | 12.5 | 96 | 83 | Current | No | No | IV |
|  | 44 | M | 19.5 | 98 | 80 | Current | No | No | IV, FC |
|  | 68 | M | 86 | 94 | 78 | Current | Yes | No | RNA, FC |
|  | 63 | M | 123 | 93 | 72 | Current | Yes | No | FC |
|  | 53 | M | 58 | 84 | 76 | Current | Yes | No | RNA |
|  | 57 | M | 25.5 | 110 | 74 | Current | No | No | FC |
|  | 55 | M | 13 | 92 | 83 | Current | No | No | FC |
|  | 41 | F | 16 | 121 | 99 | Current | No | No | FC |
|  | 66 | M | 90 | 91 | 76 | Former | Yes | No | FC |
|  | 67 | F | 15 | 85 | 85 | Former | No | No | FC |
| **Summary** | **56.8 (9.4)** | **8M/2F** | **45.8 (40.5)** | **96.4 (11.3)** | **80.6 (7.7)** | **8/2** | **4/6** | **0/11** |  |

**Supplemental Table S1. (continued)**

|  | **Age** | **Sex** | **Smoking history (pack-years)** | **FEV_1_% predicted** | **FEV_1_/FVC, %** | **Smoking Status** | **Solitary nodule (Yes/No)** | **ICS (Yes/No)** | **Experiment used** |
| --- | --- | --- | --- | --- | --- | --- | --- | --- | --- |
| **COPD** |  |  |  |  |  |  |  |  |  |
|  | 65 | M | 50 | 64 | 74 | Current | Yes | No | RNA, FC |
|  | 71 | M | 50 | 21 | 31 | Current | Yes | Yes | RNA |
|  | 65 | M | 100 | 27 | 41 | Current | Yes | Yes | RNA, IV |
|  | 61 | M | 67.5 | 42 | 50 | Current | Yes | Yes | FC |
|  | 72 | M | 61 | 49 | 47 | Current | Yes | Yes | RNA, IV, IHC |
|  | 56 | M | 86 | 53 | 66 | Current | Yes | Yes | RNA |
|  | 65 | M | 44 | 63 | 58 | Current | Yes | Yes | RNA, IV, FC |
|  | 56 | M | 80 | 75 | 66 | Current | Yes | No | IHC |
|  | 64 | M | 36 | 73 | 60 | Current | Yes | No | RNA |
|  | 57 | M | 80 | 84 | 65 | Current | Yes | No | IHC |
|  | 62 | M | 50 | 45 | 67 | Former | Yes | Yes | RNA, FC |
|  | 79 | M | 100 | 54 | 45 | Former | Yes | Yes | RNA |
|  | 70 | M | 45 | 87 | 47 | Former | No | Yes | FC |
| **Summary** | **64.8 (6.8)** | **13M/0F** | **65.3 (21.8)** | **56.7 (20.3)** | **58.1 (12.6)** | **10/3** | **12/1** | **9/4** |  |

ICS, inhaled corticosteroid use; FC, flow cytometry; IHC, immunohistochemistry. Summarized data are shown in bold and are represented as mean (SD), ratio of current smokers to former smokers, or fraction of ICS users (yes/no). All FEV1 values are pre-bronchodilator.
