# Supplementary material for: Smoking decreases the response of human lung macrophages to double-stranded RNA by reducing TLR3 expression
Source: Respir Res. 2013 Mar 9;14(1):33. doi: 10.1186/1465-9921-14-33 (PMC3599854; doi:10.1186/1465-9921-14-33)
Supplement: Additional file 2: Table S2 — Summary of all lung tissue subject demographics, smoking histories, spirometry & current smoking status. ICS, inhaled corticosteroid use. M, Male; F, Female. Summarized data are shown in bold and are represented as mean (SD), ratio of current smokers to former smokers, or fraction of ICS users (yes/no). All FEV1 values are pre-bronchodilator. [file 1465-9921-14-33-S2.doc]

**Supplemental Table S2. Summary of all lung tissue subject demographics, smoking histories, spirometry & current smoking status**

|  | **Age** | **Sex** | **Smoking history  (pack-years)** | **FEV_1_% predicted** | **FEV_1_/FVC %** | **Smoking Status** | **ICS** |
| --- | --- | --- | --- | --- | --- | --- | --- |
| **Non-COPD** | |  |  |  |  |  |  |
|  | 54 | M | 34 | 82 | 71 | Current | No |
|  | 66 | M | 49 | 117 | 75 | Current | No |
|  | 42 | F | 60 | 91 | 74 | Current | No |
|  | 68 | M | 86 | 94 | 78 | Current | No |
|  | 60 | M | 10 | 99 | 71 | Former | No |
|  | 60 | F | 35 | 87 | 86 | Former | No |
|  | 66 | M | 37.5 | 98 | 78 | Former | Yes |
| **Summary** | **54.4 (9.1)** | **5M/2F** | **44.5 (23.9)** | **95.4 (11.2)** | **76.0 (5.2)** | **4/3** | **1/6** |

**Supplemental Table S2 (continued)**

|  | **Age** | **Sex** | **Smoking history  (pack-years)** | **FEV1% predicted** | **FEV1/FVC %** | **Smoking Status** | **ICS**  **(Yes/No)** |
| --- | --- | --- | --- | --- | --- | --- | --- |
| **COPD** |  |  |  |  |  |  |  |
|  | 66 | F | 32 | 19 | 33 | Current | Yes |
|  | 61 | M | 67.5 | 42 | 50 | Current | Yes |
|  | 52 | M | 36 | 86 | 69 | Current | No |
|  | 75 | M | 183 | 70 | 56 | Current | Yes |
|  | 52 | F | 72 | 12 | 26 | Former | No |
|  | 64 | M | 67.5 | 19 | 28 | Former | Yes |
|  | 71 | F | 10.5 | 31 | 29 | Former | No |
|  | 50 | F | 80 | 19 | 23 | Former | Yes |
|  | 52 | F | 70 | 32 | 33 | Former | Yes |
|  | 55 | M | 25 | 18 | 30 | Former | Yes |
|  | 45 | M | 18.75 | 17 | 25 | Former | Yes |
|  | 59 | M | 63 | 24 | 28 | Former | Yes |
|  | 63 | M | 60 | 16 | 19 | Former | Yes |
|  | 77 | F | 58 | 52 | 47 | Former | Yes |
|  | 71 | M | 117 | 55 | 63 | Former | Yes |
|  | 62 | M | 15 | 101 | 66 | Former | No |
|  | 74 | F | 30 | 76 | 67 | Former | No |
|  | 63 | F | 80 | 64 | 73 | Former | No |
| **Summary** | **61.8 (9.4)** | **10M/8F** | **60.3 (41.4)** | **41.8 (27.7)** | **42.5 (18.6)** | **4/14** | **12/6** |

ICS, inhaled corticosteroid use. M, Male; F, Female. Summarized data are shown in bold and are represented as mean (SD), ratio of current smokers to former smokers, or fraction of ICS users (yes/no). All FEV1 values are pre-bronchodilator.
