# Supplementary figures and images for: Smoking decreases the response of human lung macrophages to double-stranded RNA by reducing TLR3 expression
Source: Respir Res. 2013 Mar 9;14(1):33. doi: 10.1186/1465-9921-14-33 (PMC3599854; doi:10.1186/1465-9921-14-33)

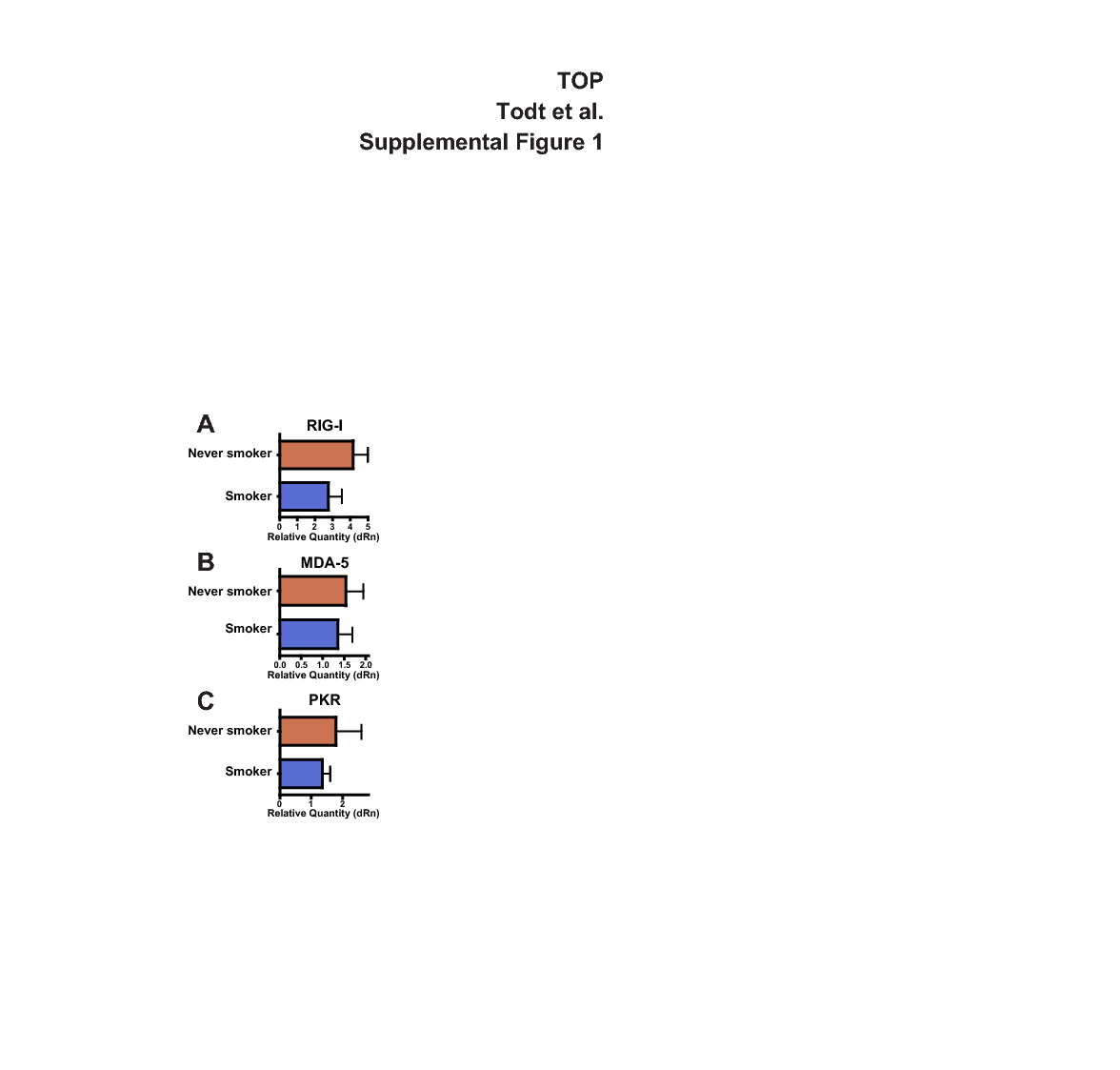

Supplement: Additional file 3: Figure S1 — AMø of current smokers show no reduction in mRNA expression of cytoplasmic dsRNA receptors, relative to AMø of never-smokers. RNA from AMø was isolated, depleted of contaminating genomic DNA, reverse-transcribed and analyzed by quantitative real-time RT-PCR using Taqman chemistry and specific primer-probe sets, normalized to GAPDH transcripts. Data are expressed on the horizontal axis as mean ± SEM for relative quantity (dRn), calculated in comparison to a single never-smoker who was arbitrarily designated the reference sample. Never-smokers (n = 6), red bars; smokers (n = 11), blue bars. The Mann–Whitney test was used to calculate statistical significance. [file 1465-9921-14-33-S3.bmp]

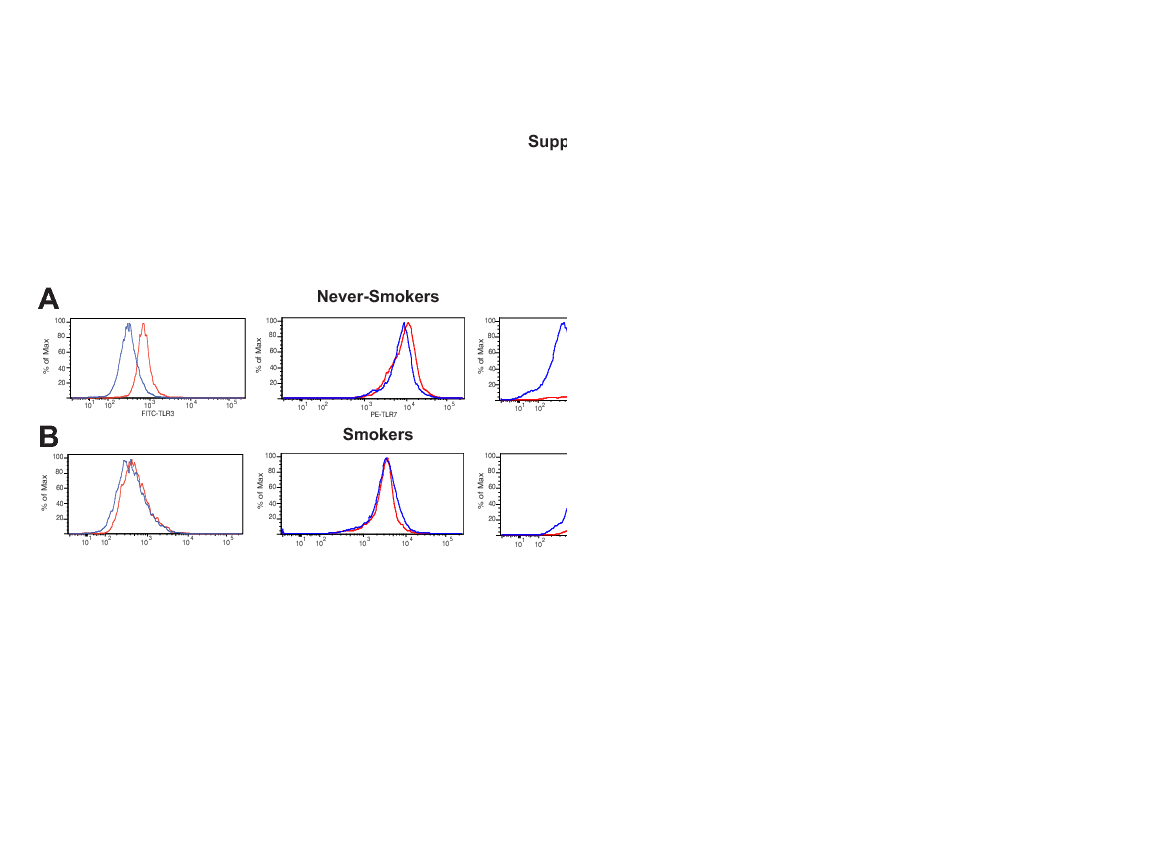

Supplement: Additional file 4: Figure S2 — Representative flow cytometry results. AMø were permeabilized, stained for expression of TLR3 (left-hand panels), TLR 7 (middle panel) and or TLR9 (right-hand panels), and analyzed by flow cytometry, gating on AMø (CD45+, high side scatter cells). A,B; specific staining (red line), isotype control staining (blue line). A, never-smoker, B, smoker. [file 1465-9921-14-33-S4.bmp]

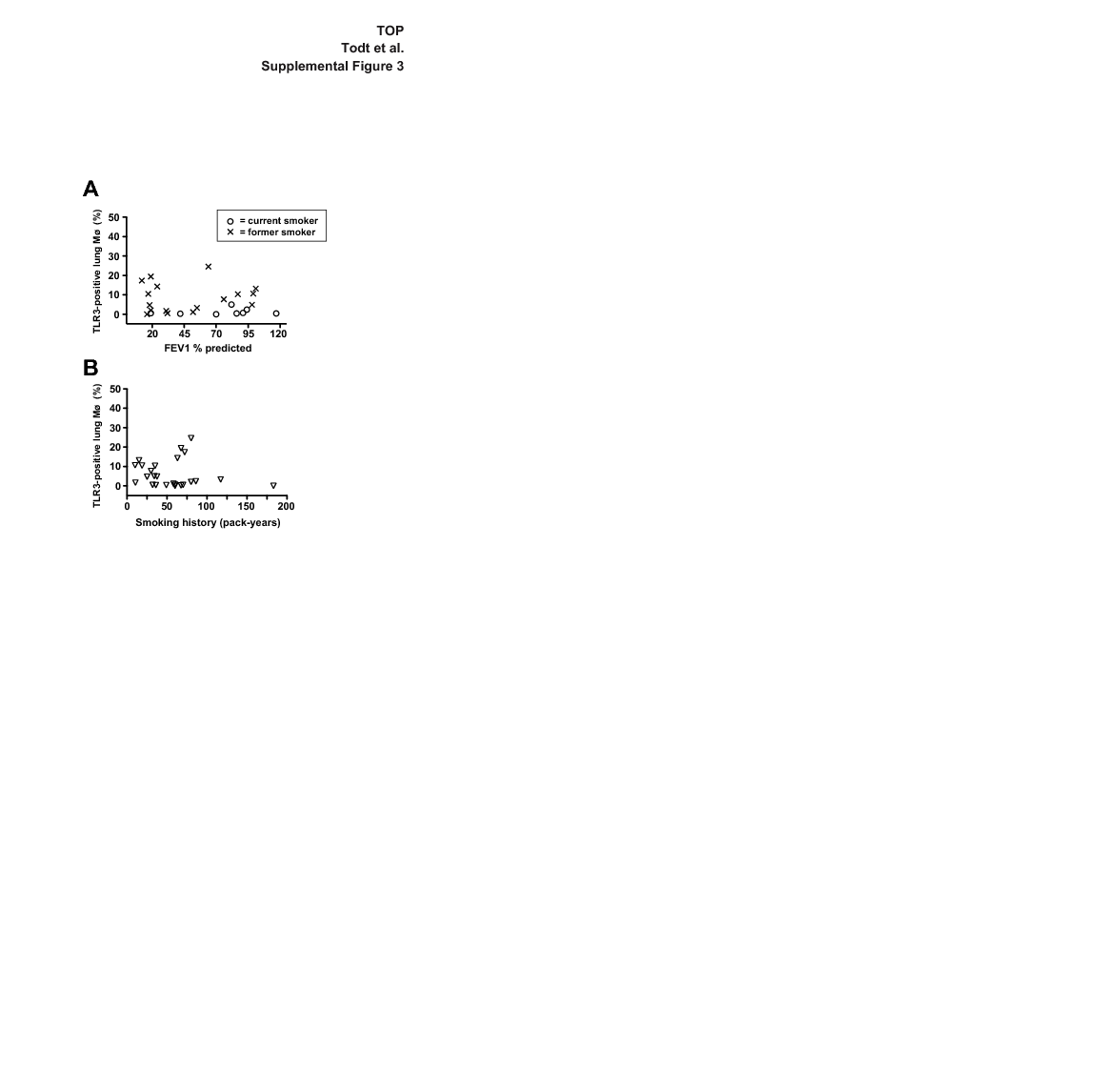

Supplement: Additional file 5: Figure S3 — Lack of correlation of spirometry or total smoking history with lung AMø TLR3 expresssion in surgical cohort. Total lung Mø were harvested from excess lung tissue removed surgically for clinical indications as decribed in the legend to Figure 3. Lung Mø were permeabilized, stained for TLR3 expression and analyzed by flow cytometry, gating on CD45+, high side-scatter cells. Data are shown as the percentage of TLR3-positive lung Mø on the vertical axis versus A, FEV1 % predicted; B, smoking history in pack-years. In panel A, circles represent current smokers and “x” respresents former smokers. In panel B, all subjects are shown as inverted triangles, regardless of smoking status (active vs. former); in both panels, n = 25. [file 1465-9921-14-33-S5.bmp]
